# Association of Urinary Concentrations of Bisphenol A and Phthalate Metabolites with Risk of Type 2 Diabetes: A Prospective Investigation in the Nurses’ Health Study (NHS) and NHSII Cohorts

**DOI:** 10.1289/ehp.1307201

**Published:** 2014-03-14

**Authors:** Qi Sun, Marilyn C. Cornelis, Mary K. Townsend, Deirdre K. Tobias, A. Heather Eliassen, Adrian A. Franke, Russ Hauser, Frank B. Hu

**Affiliations:** 1 Channing Division of Network Medicine, Department of Medicine, Brigham and Women’s Hospital and Harvard Medical School, Boston, Massachusetts, USA; 2 Department of Nutrition, and; 3 Department of Epidemiology, Harvard School of Public Health, Boston, Massachusetts, USA; 4 Natural Products and Experimental Therapeutics Program, University of Hawaii Cancer Center, Honolulu, Hawai’i, USA; 5 Department of Environmental Health, Harvard School of Public Health, Boston, Massachusetts, USA

## Abstract

Background: Prospective evidence regarding associations for exposures to bisphenol A (BPA) and phthalates with type 2 diabetes (T2D) is lacking.

Objective: We prospectively examined urinary concentrations of BPA and phthalate metabolites with T2D risk.

Methods: We measured BPA and eight major phthalate metabolites among 971 incident T2D case–control pairs from the Nurses’ Health Study (NHS) (mean age, 65.6 years) and NHSII (mean age, 45.6 years).

Results: In the NHSII, BPA levels were not associated with incident T2D in multivariate-adjusted analysis until body mass index was adjusted: odds ratio (OR) comparing extreme BPA quartiles increased from 1.40 (95% CI: 0.91, 2.15) to 2.08 (95% CI: 1.17, 3.69; *p*_trend_ = 0.02) with such an adjustment. In contrast, BPA concentrations were not associated with T2D in the NHS (OR = 0.81; 95% CI: 0.48, 1.38; *p*_trend_ = 0.45). Likewise, urinary concentrations of total phthalate metabolites were associated with T2D in the NHSII (OR comparing extreme quartiles = 2.14; 95% CI: 1.19, 3.85; *p*_trend_ = 0.02), but not in the NHS (OR = 0.87; 95% CI: 0.49, 1.53; *p*_trend_ = 0.29). Summed metabolites of butyl phthalates or di-(2-ethylhexyl) phthalates were significantly associated with T2D only in the NHSII; ORs comparing extreme quartiles were 3.16 (95% CI: 1.68, 5.95; *p*_trend_ = 0.0002) and 1.91 (95% CI: 1.04, 3.49; *p*_trend_ = 0.20), respectively.

Conclusions: These results suggest that BPA and phthalate exposures may be associated with the risk of T2D among middle-aged, but not older, women. The divergent findings between the two cohorts might be explained by menopausal status or simply by chance. Clearly, these results need to be interpreted with caution and should be replicated in future studies, ideally with multiple urine samples collected prospectively to improve the measurement of these exposures with short half-lives.

Citation: Sun Q, Cornelis MC, Townsend MK, Tobias DK, Eliassen AH, Franke AA, Hauser R, Hu FB. 2014. Association of urinary concentrations of bisphenol A and phthalate metabolites with risk of type 2 diabetes: a prospective investigation in the Nurses’ Health Study (NHS) and NHSII Cohorts. Environ Health Perspect 122:616–623; http://dx.doi.org/10.1289/ehp.1307201

## Introduction

Extensive research has established the role of lifestyle, diet, and genetic variations in the etiology of type 2 diabetes (T2D) (Qi et al. 2008). Meanwhile, emerging evidence has led to a novel hypothesis that some of these chemicals, such as bisphenol A (BPA) and phthalates, may also be related to the rising epidemics of obesity and T2D (Casals-Casas and Desvergne 2011). Both classes of chemicals are produced in large quantities worldwide and have wide industrial applications (Casals-Casas and Desvergne 2011; Hauser and Calafat 2005) and can be detected ubiquitously in human urines (Calafat et al. 2008; Silva et al. 2004).

Animal experiments suggest that, in addition to its well-known estrogenic effects, BPA may also interfere with multiple pathways related to T2D, including impaired beta-cell function (Alonso-Magdalena et al. 2006), liver dysfunction (Bindhumol et al. 2003; Nakagawa and Tayama 2000), dysregulation of glucose metabolism and adiponectin release in adipocytes (Ben-Jonathan et al. 2009; Hugo et al. 2008), and disruption of thyroid hormone functions (Moriyama et al. 2002). Experimental evidence suggests that phthalates may also affect the liver and interfere with adipocyte biology and glucose metabolism through effects on peroxisome proliferator-activated receptors (PPARs) (Desvergne et al. 2009). Despite the accumulation of evidence from animal studies, evidence among humans for associations of BPA and phthalates with T2D has been limited to cross-sectional studies, with mixed findings (James-Todd et al. 2012; LaKind et al. 2012; Lang et al. 2008; Lind et al. 2012b; Ning et al. 2011; Shankar and Teppala 2011; Silver et al. 2011; Svensson et al. 2011).

Therefore, we analyzed data from two prospective cohort studies among U.S. women, the Nurses’ Health Study (NHS) and the NHSII, to evaluate associations between urinary concentrations of BPA and phthalate metabolites and incident T2D. The non-overlapping age distributions between the two cohorts allowed us to examine these associations among different age groups and by menopausal status. Based on experimental data suggesting that BPA interferes with beta-cell functions by activating estrogen receptors (Alonso-Magdalena et al. 2006; Nadal et al. 2009; Soriano et al. 2012), we hypothesized that associations with BPA would be stronger in premenopausal women than in postmenopausal women.

## Methods

*Study population*. The NHS was established in 1976 when 121,700 female registered nurses 30–55 years of age were enrolled, whereas in 1989 the younger counterpart NHSII cohort was initiated among a total of 116,430 female registered nurses 25–42 years of age. A total of 18,743 NHS participants 53–79 years of age provided blood and urine samples in 2000–2002. In 1996–2001, blood and urine samples were collected from 29,611 NHSII participants 32–52 years of age. Urine samples were collected without preservative in a polypropylene container and returned to a central biorepository via overnight courier with an icepack, where they were processed immediately upon arrival and aliquoted into polypropylene cryovials, which were stored in the vapor phase of liquid nitrogen freezers at ≤ –130°C. In both cohorts, a high follow-up rate of > 90% was maintained among participants who provided urine samples.

*Assessment of covariates*. NHS participants responded to a questionnaire inquiring about body weight, height, demographic and lifestyle information, and medical history at study baseline. Similar follow-up questionnaires have been administered biennially since baseline to update these variables. In 1984, 1986, and every 4 years thereafter, a validated food frequency questionnaire (FFQ) has been used to assess participants’ usual diet. In the NHSII, questionnaires similar to those used in the NHS are sent biennially to update lifestyle and health-related characteristics. The FFQ was first administered in 1991 and is updated every 4 years in the NHSII. Based on the FFQ, we derived a score of the Alternative Healthy Eating Index (AHEI), an indicator of adherence to healthy eating behavior (McCullough et al. 2002). Of note, the FFQs did not inquire about any food packaging information. Information on cigarette smoking, physical activity, family history of diabetes, menopausal status, oral contraceptive use, hormone replacement therapy, and the history of hypertension or hypercholesterolemia were also assessed in the questionnaires at baseline and during follow-up. In the questionnaires, participants were asked about the average time per week in the past year spent on leisure-time physical activities. Based on this information, we calculated energy expenditure in metabolic equivalent tasks (METs) measured in hours per week.

*Ascertainment of T2D*. At baseline and on all biennial follow-up questionnaires, participants were asked whether they had received a physician-diagnosis of diabetes. Those reporting a diabetes diagnosis were sent a supplementary questionnaire (Manson et al. 1991) regarding any symptoms, diagnostic tests, and treatment. We used one of the following American Diabetes Association 1998 criteria to confirm self-reported T2D diagnosis: *a*) an elevated glucose concentration (fasting plasma glucose ≥ 7.0 mmol/L, random plasma glucose ≥ 11.1 mmol/L, or plasma glucose ≥ 11.1 mmol/L after an oral glucose load) and at least one symptom (excessive thirst, polyuria, weight loss, or hunger) related to diabetes; *b*) no symptoms, but elevated glucose concentrations on two separate occasions; or *c*) treatment with insulin or oral hypoglycemic medication. The accuracy of self-reported diagnosis of T2D has been demonstrated in a validation study (Manson et al. 1991), in which a blinded endocrinologist confirmed the diagnosis of diabetes by reviewing medical records of 61 of 62 NHS participants who responded to the supplementary questionnaire. Only confirmed T2D cases were included in the present study in order to minimize possible misclassification.

*Nested case–control study*. We prospectively identified and confirmed 971 T2D cases (NHS, 394; NHSII, 577) through June 2008 (NHS) or June 2007 (NHSII) among participants who provided first morning urine samples and were free of T2D, cardiovascular disease, and major cancers except nonmelanoma skin cancer at sample collection. Using the risk–set sampling scheme, we randomly selected one control for each case from among the women who remained free of T2D at the case’s date of diagnosis (Prentice and Breslow 1978). We matched cases and controls for age at urine sample collection (± 1 year), date (± 3 months)/time (first morning or not) of sample collection, ethnicity (white or other), fasting status when blood was drawn (≥ 8 hr or not), and menopausal status (yes, no) and hormone replacement therapy use (yes, no) at sample collection (NHSII only). T2D cases diagnosed within the first year since urine sample collection were excluded from selection in order to reduce the potential for reverse causation bias.

The study protocol was approved by the institutional review board of the Brigham and Women’s Hospital and the Human Subjects Committee Review Board of Harvard School of Public Health. Informed consent was provided by all participants involved in this research.

*Laboratory measurements*. Urinary concentrations of BPA and eight phthalate metabolites [mono-(2-ethylhexyl) phthalate (MEHP), mono-(2-ethyl-5-hydroxyhexyl) phthalate (MEHHP), mono(2-ethyl-5-carboxypentyl) phthalate (MECPP), mono-(2-ethyl-5-oxohexyl) phthalate (MEOHP), monobutyl phthalate (MBP), mono-isobutyl phthalate (MiBP), monobenzyl phthalate (MBzP), monoethyl phthalate (MEP), and phthalic acid] were measured using established methods with modifications (Fox et al. 2011; Kato et al. 2005) (see Supplemental Material, “Laboratory measurements,” pp. 2–3, for complete details). Briefly, samples were mixed with isotopically-labeled phthalate metabolites and BPA and treated with β-glucuronidase and sulfatase. Urinary concentrations of phthalate metabolites were measured by orbitrap-liquid chromatography–mass spectrometry (LCMS) (model Exactive; Thermo Electron, Waltham, MA), and BPA concentrations were measured by tandem-LCMS (model TSQ Ultra; Thermo Electron) at A. Franke’s laboratory at the University of Hawaii Cancer Center in 2012. Of note in the NHS only, because of technical reasons, concentrations of phthalic acid were not available for 144 case–control pairs, and only combined concentrations of MBP and MiBP were available. We also measured urinary creatinine levels using a Roche-Cobas MiraPlus clinical chemistry autoanalyzer (Roche Diagnostics, Indianapolis, IN) with a kit from Randox Laboratories (Crumlin, UK). Lastly, In the NHS we measured two liver enzymes [alanine transaminase (ALT) and γ-glutamyl transpeptidase (GGT)] using a direct enzymatic colorimetric assay, performed on the Roche P Modular system (Roche Diagnostics). In addition, fetuin-A levels were measured by an enzyme immunoassay from R&D Systems (Minneapolis, MN) in the NHS (Sun et al. 2013).

*Quality control procedures*. Each pair of matched case–control urine samples was shipped in the same batch and analyzed in the same run. Within each batch, samples were assayed by the same technician in a random sequence under identical conditions. Duplicates of blinded quality control samples (*n* = 82 for NHS; 116 for NHSII) were run along with the case–control samples to monitor the quality of these assays. We calculated intra-assay coefficients of variation (CVs) based on the measurements of these samples. The average CVs were < 10% for most metabolites (including creatinine), except for MEHP (NHS 11.4%, NHSII 10.0%) and BPA (NHS 11.5%, NHSII 13.0%).

*Within-person stability of metabolites*. We measured BPA and phthalates in two urine samples collected 1–3 years apart from a separate sample of 120 participants to evaluate the within-person reproducibility (Townsend et al. 2013). The creatinine-adjusted intraclass correlation coefficients (ICCs) between the two measurements were ≥ 0.30 for all metabolites [ranging from 0.30 for MiBP to 0.53 for MBP, except for MEHP and BPA (0.14 for both)].

*Statistical methods*. We summed molar concentrations of MEHP, MEHHP, MECPP, and MEOHP to represent total metabolites of di(2-ethylhexyl) phthalate (DEHP). Likewise, we summed molar concentrations of MBP and MiBP. We calculated total phthalate concentrations (in nanomoles per liter) as the summed values of MEHP, MEHHP, MECPP, MEOHP, MBP, MiBP, MBzP, and MEP to facilitate comparisons with previous studies. We calculated Spearman correlation coefficients (*r*_S_) among controls to evaluate the intercorrelation among urinary metabolites.

Study participants were categorized into quartiles according to the cohort-specific distribution of metabolite concentrations among controls. We used conditional logistic regression to model the associations under investigation. We adjusted for body mass index (BMI; < 25.0 kg/m^2^, 25.0–27.4 kg/m^2^, 27.5–29.9 kg/m^2^, 30.0–32.4 kg/m^2^, ≥ 32.5 kg/m^2^, missing category), smoking status (current smoker, past smoker, nonsmoker), oral contraceptive use (never used, past user, current user; NHSII only), hormone replacement therapy (yes, no; NHS only), physical activity (METs-hr/week), alcohol use (abstainer, < 5.0 g/day, 5.0–14.9 g/day, ≥ 15.0 g/day), family history of diabetes (yes, no), history of hypercholesterolemia or hypertension (yes, no), AHEI score, and urinary creatinine (mg/dL). In the current analysis we used measurements of covariates derived from the questionnaire administered in 2000 (1998 for AHEI and alcohol use) in NHS or 1995 in NHSII. *p*-Values for linear trend were calculated by modeling the median value of each quartile as a continuous variable. We pooled cohort-specific estimates for the NHS and NHSII using a random-effects meta-analysis. Heterogeneity of odds ratios (ORs) between the two cohorts was evaluated by the Cochrane Q statistic and the *I*^2^ statistic.

Restricted cubic spline regressions with 3 knots were used to model potential dose–response relations between the metabolites and diabetes (Durrleman and Simon 1989). In this analysis, to maximize statistical power, we pooled data for case–control pairs from the two cohorts and then performed statistical analyses using conditional logistic regression. In addition, participants in the top 5% of metabolite concentrations were excluded to minimize the potential impact of extreme outliers. We used likelihood ratio tests (LRTs) to examine nonlinearity, comparing the model with the linear term only to the model with the linear plus the cubic spline terms.

LRTs were also used to examine effect modification of associations between metabolites and T2D by testing the significance of multiplicative interaction terms between metabolite quartiles modeled as ordinal variables and each potential modifier.

As secondary analyses, we calculated Spearman partial correlation coefficients between the chemicals and plasma levels of liver enzymes and fetuin-A, as well as their interactions on incident T2D, in the NHS to evaluate the potential adverse effects of the pollutants on liver function as suggested by animal experiments and human observational studies (Desvergne et al. 2009; Lang et al. 2008).

All *p*-values were two-sided and *p* < 0.05 was considered statistical significance. Data were analyzed with SAS, version 9.2 (SAS Institute Inc., Cary, NC).

## Results

Table 1 shows the characteristics of the cases and controls at sample collection. The NHS participants were, on average, 20 years older than the NHSII counterparts at urine sample collection. Differences in menopausal status are consistent with differences in age between the two cohorts. Urinary concentrations of BPA and phthalate metabolites were higher in NHSII participants than NHS participants. In both cohorts, cases had higher urinary concentrations of DEHP metabolites than controls, whereas the concentrations of other chemicals were similar.

**Table 1:** Characteristics of diabetes cases and controls in the NHS and NHSII at urinary sample collection.

Characteristic^*a*^	NHS			NHSII		
	Cases (*n* = 394)	Controls (*n* = 393)	*p*-Value^*b*^	Cases (*n* = 577)	Controls (*n* = 577)	*p*-Value^*b*^
Age at urine sample collection (years)^*c*^	65.6 ± 6.4	65.6 ± 6.4	0.98	45.6 ± 4.4	45.6 ± 4.4	0.91
BMI (kg/m^2^)^*d*^	29.7 ± 5.8	26.0 ± 4.5	< 0.0001	33.5 ± 7.0	25.7 ± 5.5	< 0.0001
BMI categories (%)			< 0.0001			< 0.0001
< 25.0	21.3	47.8		9.7	54.6
25.0–27.4	16.5	20.1		11.4	16.1
27.5–29.9	18.5	12.7		11.3	8.8
30.0–32.4	14.7	9.4		14.6	5.9
≥ 32.5	27.4	9.4		49.1	10.6
Missing	1.5	0.5		4.0	4.0
Physical activity (MET-hr/week)	16.8 ± 22.8	19.0 ± 20.9	0.15	17.0 ± 27.3	19.5 ± 22.9	0.09
Smoking status (%)			0.58			0.02
Current smoker	6.6	5.9		12.7	8.2
Former smoker	47.5	44.5		22.9	27.4
Never smoked	45.9	49.6		64.5	64.5
Alcohol intake (%)			0.01			0.0002
Abstainer	25.1	20.6		42.5	32.6
< 5.0 g/day	50.0	45.8		46.3	48.4
5.0–14.9 g/day	15.2	24.4		8.5	14.7
≥ 15.0 g/day	9.6	9.2		2.8	4.3
Hypertension (%)	61.9	40.0	< 0.0001	27.6	12.5	< 0.0001
Hypercholesterolemia (%)	73.4	59.5	< 0.0001	46.5	23.2	< 0.0001
White (%)^*c*^	97.7	98.2	0.62	95.7	96.5	0.45
Family history of diabetes (%)	38.8	27.5	0.0007	32.6	16.6	< 0.0001
Fasting ≥ 8 hr at blood sample collection (%)^*c*^	93.2	94.2	0.57	73.8	75.9	0.42
Menopause (%)^*c*^	100.0	99.2	0.25	34.7	34.7	> 0.99
Postmenopausal hormone use (%)^*c*^^,^^*e*^	59.1	62.1	0.40	49.0	49.0	> 0.99
Use of oral contraceptive (%)^*f*^			—			0.05
Current user	—	—		2.3	4.9
Past user	—	—		84.2	83.4
Never used	—	—		13.5	11.8
Diet
Total energy (kcal/day)	1778.9 ± 415.4	1761.2 ± 402.0	0.54	1861.1 ± 513.2	1784.0 ± 485.7	0.009
Trans fats (% of energy)	1.63 ± 0.40	1.58 ± 0.40	0.07	1.61 ± 0.52	1.51 ± 0.49	0.0006
Polyunsaturated: saturated fat ratio	0.56 ± 0.12	0.58 ± 0.14	0.05	0.50 ± 0.12	0.52 ± 0.14	0.008
Coffee (cups/day)	2.0 ± 1.4	2.2 ± 1.5	0.09	1.3 ± 1.7	1.7 ± 1.7	0.0003
Whole grains (g/day)	18.6 ± 10.3	20.7 ± 10.0	0.004	19.6 ± 12.8	22.3 ± 14.4	0.0008
Fruits (servings/day)	2.2 ± 1	2.3 ± 1.0	0.07	1.8 ± 1.1	1.9 ± 1.3	0.01
Vegetables (servings/day)	3.2 ± 1.3	3.3 ± 1.4	0.24	2.8 ± 1.6	2.8 ± 1.8	0.85
Red meat (servings/day)	0.9 ± 0.4	0.8 ± 0.4	< 0.0001	0.9 ± 0.6	0.8 ± 0.6	< 0.0001
Fish (servings/day)	0.3 ± 0.2	0.3 ± 0.2	0.94	0.2 ± 0.2	0.2 ± 0.2	0.29
Soft drinks (servings/day)	0.8 ± 0.8	0.6 ± 0.7	0.0004	1.7 ± 1.5	1.2 ± 1.2	< 0.0001
AHEI score^*g*^	50.0 ± 8.2	52.7 ± 8.6	< 0.0001	46.7 ± 9.2	49.5 ± 9.8	< 0.0001
Urinary creatinine (mg/dL)^*h*^	66.6 (45.2, 101.6)	60.9 (43.0, 90.9)	0.05	87.8 (63.9, 127.1)	87.8 (61.5, 119.6)	0.25
Urinary BPA (μg/L)^*h*^	1.5 (1.0, 2.8)	1.5 (1.0, 2.7)	0.98	2.3 (1.4, 3.8)	2.0 (1.3, 3.5)	0.19
Urinary phthalate metabolites (nmol/L)^*h*^
DEHP^*i*^	277.6 (154.4, 545.8)	229.7 (142.8, 463.7)	0.05	324.7 (201.4, 586.3)	301.7 (170.8, 522.3)	0.02
Butyl phthalates^*i*^	107.7 (70.8, 209.8)	114.8 (67.8, 212.0)	0.88	249.2 (151, 421.0)	248.5 (150.6, 378.4)	0.44
Total phthalates^*i*^	1055.1 (616.1, 1885.3)	1049.6 (571.8, 2010.3)	0.87	1495.6 (900.8, 2876.6)	1479.5 (858.5, 2684.2)	0.32
^***a***^Plus-minus values are mean ± SD. Percentages are based on non-missing data. ^***b***^*p*-Value estimates are based on Student’s *t*-test for variables expressed as mean ± SD, Wilcoxon rank–sum test for variables expressed as median (IQR), or Pearson χ^2^ test for variables expressed as percentages. ^***c***^Menopausal status and hormone use were matching factors in the NHSII only. ^***d***^BMI was missing for 8 participants in the NHS and 46 participants in the NHSII. ^***e***^Among menopausal women only. ^***f***^Among premenopausal women only. ^***g***^Measuring the overall diet quality by summarizing higher intakes of vegetables, fruits, nuts, soy, and cereal fiber, higher ratios of chicken plus fish to red meat and polyunsaturated to saturated fat, lower intake of *trans* fats, and multivitamin use of ≥ 5 years. ^***h***^Values are median (25th percentile, 75th percentile). ^***i***^DEHP metabolites included MEHP, MEHHP, MEOHP, and MECPP; butyl phthalate metabolites, MBP and MiBP; total phthalate metabolites, MEP, MEHP, MEHHP, MECPP, MEOHP, MBzP, MBP, and MiBP.						

Phthalate metabolites derived from the same parent chemicals were strongly correlated with each other (*r*_S_ = 0.59–0.91 among DEHP metabolites, or 0.98 between the two butyl phthalate metabolites), and correlations were weaker otherwise (see Supplemental Material, Table S1). BPA concentrations were correlated with some of the phthalate metabolites, although the correlations were weak to moderate (*r*_S_ ≤ 0.26).

Estimated associations between BPA concentrations and incident T2D are shown in Table 2. In both cohorts, BPA was not associated with T2D based on conditional logistic models that accounted for matching and were adjusted for creatinine levels only (model 1). In the NHS, further adjustment for covariates (model 2), and additional adjustment for BMI (model 3), did not change associations with BPA materially. In contrast, in the NHSII, we estimated significant positive associations with T2D after adjustment for additional covariates, especially BMI (model 3; OR = 2.08; 95% CI: 1.17, 3.69; *p*_trend_ = 0.02).

**Table 2:** ORs (95% CIs) of incident T2D by quartiles of urinary concentrations (μg/L) of BPA: the NHS and NHSII.

Variable	Quartile 1 (lowest)	Quartile 2	Quartile 3	Quartile 4 (highest)	*p*_trend_
NHS/BPA
Median (range)	0.7 (0.03–1.0)	1.2 (1.0–1.5)	2.0 (1.5–2.7)	4.4 (2.8–56.1)
Case/control (*n*)	102/99	95/98	98/98	99/98
Model 1^*a*^	1	0.92 (0.62, 1.37)	0.85 (0.56, 1.29)	0.81 (0.52, 1.26)	0.40
Model 2^*b*^	1	0.99 (0.63, 1.54)	0.96 (0.60, 1.53)	0.77 (0.47, 1.26)	0.25
Model 3^*c*^	1	0.91 (0.56, 1.48)	0.98 (0.60, 1.61)	0.81 (0.48, 1.38)	0.45
NHSII/BPA
Median (range)	0.9 (0.03–1.3)	1.7 (1.3–2.0)	2.7 (2.0–3.5)	5.4 (3.5–45.4)
Case/control (*n*)	132/144	132/144	154/145	159/144
Model 1^*a*^	1	0.97 (0.69, 1.37)	1.09 (0.77, 1.54)	1.10 (0.76, 1.59)	0.54
Model 2^*b*^	1	1.08 (0.72, 1.61)	1.29 (0.86, 1.94)	1.40 (0.91, 2.15)	0.13
Model 3^*c*^	1	1.34 (0.70, 2.27)	1.91 (1.11, 3.29)	2.08 (1.17, 3.69)	0.02
^***a***^In model 1, we automatically adjusted for matching factors, including age at urine sample collection, ethnicity, fasting status, and time of sample collection, menopausal status, and use of hormone replacement therapy (NHSII only), by using conditional logistic regression, and also for urinary creatinine levels. ^***b***^Based on model 1, we adjusted model 2 for smoking status, postmenopausal hormone use (NHS only), oral contraceptive use (NHSII only), physical activity, alcohol use, family history of diabetes, history of hypercholesterolemia or hypertension, and AHEI score. ^***c***^Based on model 2, we adjusted model 3 for BMI.					

We observed a similar pattern of divergent associations between two cohorts for summed DEHP, butyl, and total phthalate metabolites (Table 3). In the NHSII, butyl phthalate and total phthalate metabolites were significantly associated with T2D. After adjusting for confounders, especially BMI, ORs comparing extreme quartiles for the butyl and total phthalate metabolites were 3.16 (95% CI: 1.68, 5.95; *p*_trend_ = 0.0002) and 2.14 (95% CI: 1.19, 3.85; *p*_trend_ = 0.02), respectively.

**Table 3:** ORs (95% CIs) of incident T2D by quartiles of urinary concentrations of phthalate metabolites (nmol/L): the NHS and NHSII.

Variable	Quartile 1 (lowest)	Quartile 2	Quartile 3	Quartile 4 (highest)	*p*_trend_
NHS
DEHP^*a*^
Median (range)	102.9 (1.5–142.8)	180.1 (142.8–229.7)	311.6 (231.8–463.7)	844.5 (464.5–27829.8)
Case/control (*n*)	88/98	79/99	104/98	123/98
Model 1^*b*^	1	0.85 (0.54, 1.32)	1.09 (0.71, 1.66)	1.27 (0.81, 1.99)	0.13
Model 2^*c*^	1	0.82 (0.50, 1.35)	1.06 (0.65, 1.72)	1.34 (0.80, 2.22)	0.10
Model 3^*d*^	1	0.88 (0.52, 1.50)	1.02 (0.61, 1.71)	1.34 (0.77, 2.30)	0.14
Butyl phthalates^*a*^
Median (range)	47.1 (1.6–67.6)	88.7 (67.8–113.2)	152.0 (114.3–212.0)	334.2 (213.2–12702.7)
Case/control (*n*)	96/98	109/97	94/100	95/98
Model 1^*b*^	1	1.06 (0.69, 1.63)	0.80 (0.51, 1.26)	0.72 (0.43, 1.20)	0.15
Model 2^*c*^	1	1.20 (0.74, 1.96)	0.93 (0.56, 1.55)	0.94 (0.53, 1.68)	0.65
Model 3^*d*^	1	1.26 (0.75, 2.12)	1.01 (0.59, 1.73)	0.91 (0.50, 1.68)	0.51
Total phthalates^*a*^
Median (range)	391.6 (11.0–571.4)	770.5 (571.8–1049.3)	1386.5 (1049.6–2010.3)	3824.1 (2010.6–49621.3)
Case/control (*n*)	89/98	107/98	105/99	93/98
Model 1^*b*^	1	1.13 (0.74, 1.71)	1.07 (0.70, 1.63)	0.86 (0.54, 1.38)	0.27
Model 2^*c*^	1	1.20 (0.75, 1.91)	1.07 (0.67, 1.71)	0.90 (0.53, 1.53)	0.36
Model 3^*d*^	1	1.20 (0.72, 1.99)	1.15 (0.70, 1.91)	0.87 (0.49, 1.53)	0.29
NHSII
DEHP^*a*^
Median (range)	123.2 (1.5–170.4)	223.0 (170.8–300.1)	376.8 (301.7–522.3)	869.7 (525.7–11707.2)
Case/control (*n*)	99/144	165/144	139/145	174/144
Model 1^*b*^	1	1.64 (1.16, 2.31)	1.36 (0.95, 1.96)	1.72 (1.16, 2.54)	0.08
Model 2^*c*^	1	1.67 (1.12, 2.49)	1.47 (0.96, 2.24)	1.89 (1.20, 2.98)	0.05
Model 3^*d*^	1	1.80 (1.07, 3.04)	1.62 (0.95, 2.76)	1.91 (1.04, 3.49)	0.20
Butyl phthalates^*a*^
Median (range)	107.0 (1.6–150.4)	199.5 (150.6–248.0)	300.3 (248.5–378.4)	591.0 (379.3–29543.2)
Case/control (*n*)	144/144	144/144	112/145	177/144
Model 1^*b*^	1	0.95 (0.68, 1.35)	0.70 (0.48, 1.03)	1.08 (0.74, 1.60)	0.34
Model 2^*c*^	1	1.00 (0.67, 1.49)	0.82 (0.52, 1.27)	1.48 (0.94, 2.33)	0.03
Model 3^*d*^	1	1.38 (0.81, 2.35)	1.17 (0.66, 2.10)	3.16 (1.68, 5.95)	0.0002
Total phthalates^*a*^
Median (range)	599.8 (11.0–858.5)	1104.2 (858.5–1462.9)	1878.2 (1479.5–2684.2)	4348.5 (2695.5–60068.1)
Case/control (*n*)	143/144	156/144	117/145	161/144
Model 1^*b*^	1	1.10 (0.79, 1.55)	0.89 (0.61, 1.29)	1.09 (0.75, 1.58)	0.64
Model 2^*c*^	1	1.25 (0.83, 1.89)	0.97 (0.62, 1.50)	1.27 (0.82, 1.96)	0.36
Model 3^*d*^	1	1.73 (1.01, 2.97)	1.08 (0.61, 1.92)	2.14 (1.19, 3.85)	0.02
^***a***^DEHP metabolites included MEHP, MEHHP, MEOHP, and MECPP; butyl phthalates, MBP and MiBP; total phthalates, MEP, MEHP, MEHHP, MECPP, MEOHP, MBzP, MBP, and MiBP. ^***b***^In model 1, we automatically adjusted for matching factors, including age at urine sample collection, ethnicity, fasting status, and time of sample collection, menopausal status, and use of hormone replacement therapy (NHSII only), by using conditional logistic regression, and also for urinary creatinine levels. ^***c***^Based on model 1, we adjusted model 2 for smoking status, postmenopausal hormone use (NHS only), oral contraceptive use (NHSII only), physical activity, alcohol use, family history of diabetes, history of hypercholesterolemia or hypertension, and AHEI score. ^***d***^Based on model 2, we adjusted model 3 for BMI.					

Analysis on joint associations between BPA and butyl phthalates in the NHSII showed that the effects of these two classes of compounds were multiplicative (*p*_interaction_ = 0.96) (see Supplemental Material, Figure S1). Furthermore, we did not observe any significant correlations between the chemicals and liver enzymes or fetuin-A levels measured in the NHS (*r*_S_ ≤ 0.10), or any significant effect modifications by liver enzymes on the associations between the pollutants and T2D risk (data not shown).

**Figure 1:**
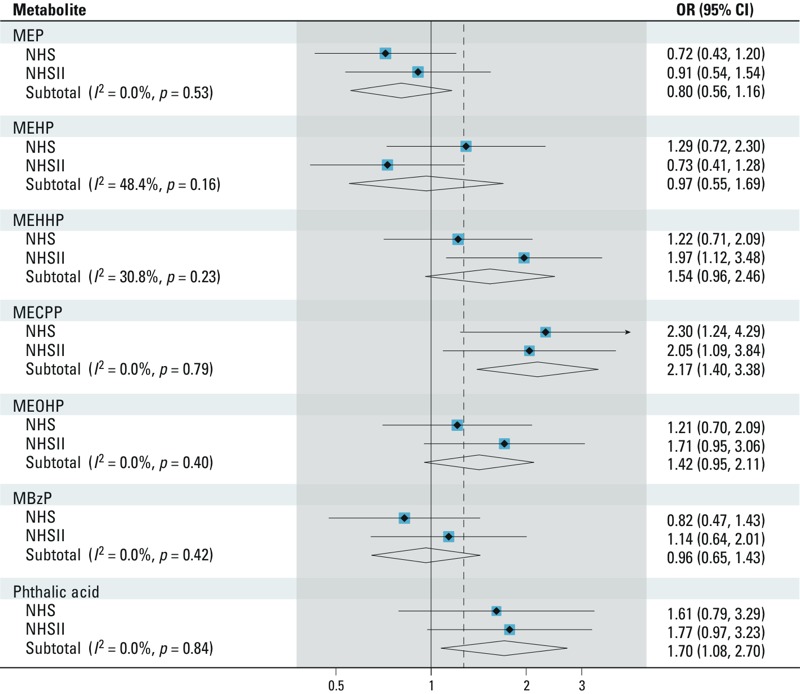
Pooled ORs (95% CIs) of incident T2D comparing extreme quartiles of individual urinary concentrations of phthalate metabolites in the NHS and NHSII. We adjusted the conditional logistic regression model for matching factors, including age at urine sample collection, ethnicity, fasting status, and time of sample collection, menopausal status, and use of hormone replacement therapy (NHSII only), urinary creatinine levels, BMI, smoking status, postmenopausal hormone use (NHS only), oral contraceptive use (NHSII only), physical activity, alcohol use, family history of diabetes, history of hypercholesterolemia or hypertension, and AHEI score.

The associations between individual phthalate metabolites and incident T2D are presented in Supplemental Material, Table S2. In the NHSII, both individual butyl phthalate metabolites (MBP and MiBP) were positively associated with T2D. When cohort estimates were combined, we observed significant associations for MECPP and phthalic acid (Figure 1); the pooled ORs comparing extreme quartiles were 2.17 (95% CI: 1.40, 3.38; *p*_heterogeneity_ = 0.79) and 1.70 (95% CI: 1.08, 2.70; *p*_heterogeneity_ = 0.84), respectively.

**Figure 2:**
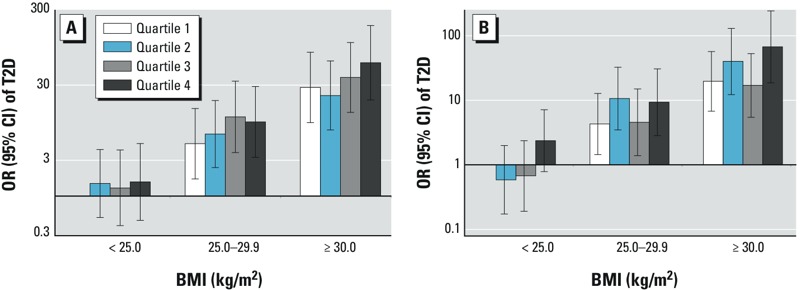
The associations of BPA and butyl phthalates with incident T2D by BMI at urine sample collection, the NHSII. (A) Joint associations between BPA and BMI; (B) joint associations between butyl phthalates and BMI. Data were analyzed using conditional logistic regression to examine joint associations between the chemicals and BMI. We adjusted these analyses for matching factors, including age at urine sample collection, ethnicity, fasting status, and time of sample collection, menopausal status and use of hormone replacement therapy (NHSII only), urinary creatinine levels, smoking status, postmenopausal hormone use (NHS only), oral contraceptive use (NHSII only), physical activity, alcohol use, family history of diabetes, history of hypercholesterolemia or hypertension, and AHEI score.

We conducted an ad hoc analysis to explore whether the positive associations of BPA and butyl phthalates in the NHSII were entirely due to the confounding by BMI. Specifically, we examined joint associations between BMI and BPA or butyl phthalate levels using conditional logistic regression with multivariate adjustment of all covariates except BMI. In general, within each BMI category, the highest BPA or butyl phthalate quartile tended to be associated with higher T2D risk in comparison with the lowest quartile (Figure 2; *p*_interaction_ ≥ 0.64). Of note, participants with missing BMI values (4%) were excluded from this analysis.

We explored age and age-related factors that may explain the heterogeneous associations of BPA between the two cohorts. Using combined data from both cohorts, we found that the association of BPA was stronger for diabetes cases occurred at a relatively younger age (≤ 55 years) than older cases (data not shown). Furthermore, the positive association between BPA and T2D risk was primarily ascribed to the menopausal status of the cases at time of diagnosis (Table 4). The ORs comparing highest versus lowest quartiles were 5.83 (95% CI: 1.68, 20.19; *p*_trend_ = 0.01) for premenopausal cases and 0.83 (95% CI: 0.54, 1.27; *p*_trend_ = 0.50) for postmenopausal cases (*p*_interaction_ = 0.03). Using the NHSII data only, we observed a similar pattern of associations, although the test for interaction was not significant: the corresponding ORs were 4.08 (95% CI: 1.39, 12.0; *p*_trend_ = 0.01) and 1.29 (95% CI: 0.62, 2.68; *p*_trend_ = 0.56), respectively. We did not observe the same interactions for phthalates (data not shown). We also explored whether the stronger associations observed in the NHSII may be due to higher concentrations of the pollutants in this younger cohort by categorizing the NHSII participants using the cut points for making quartiles in the NHS. The associations did not change materially in this analysis (data not shown).

**Table 4:** ORs (95% CIs) of incident T2D by quartiles of urinary concentrations of BPA according to the menopausal status of cases: the NHS and NHSII.

Menopausal status at diabetes diagnosis	Quartile 1 (lowest)	Quartile 2	Quartile 3	Quartile 4 (highest)	*p*_trend_	*p*_interaction_
Premenopause
Case/control (*n*)	39/45	54/55	77/79	80/71
Model 1^*a*^	1.0	2.53 (0.82, 7.76)	3.39 (1.14, 10.09)	5.83 (1.68, 20.19)	0.01	0.03
Postmenopause
Case/control (*n*)	194/197	191/188	162/162	174/172		
Model 1^*a*^	1.0	0.85 (0.59, 1.23)	0.85 (0.57, 1.27)	0.83 (0.54, 1.27)	0.50	
^***a***^Adjusted for matching factors, BMI, smoking status, postmenopausal hormone use (NHS only), oral contraceptive use (NHSII only), physical activity, alcohol use, family history of diabetes, history of hypercholesterolemia or hypertension, urinary creatinine levels, and AHEI score.						

**Figure 3:**
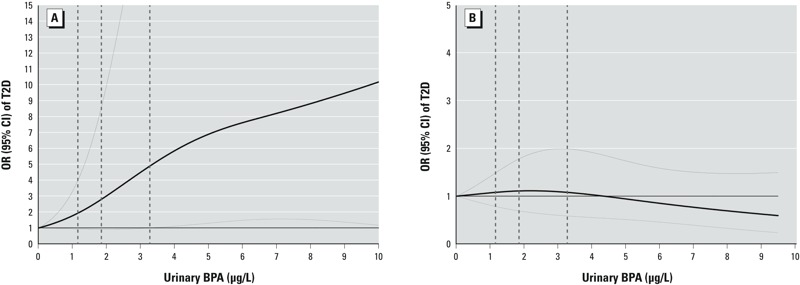
ORs (95% CIs) of incident T2D by urinary concentrations of BPA. (*A*) Among premenopausal diabetes cases and their matched controls; (*B*) among postmenopausal diabetes cases and their matched controls. Study participants with the highest 5% of BPA levels were excluded to minimize potential impact of outliers. We adjusted the multivariate conditional logistic regression models with restricted cubic splines for matching factors, including age at urine sample collection, ethnicity, fasting status, and time of sample collection, menopausal status and use of hormone replacement therapy (NHSII only), urinary creatinine levels, BMI, smoking status, postmenopausal hormone use (NHS only), oral contraceptive use (NHSII only), physical activity, alcohol use, family history of diabetes, history of hypercholesterolemia or hypertension, and AHEI score. Solid lines are ORs and dashed lines are 95% CIs. The dotted vertical lines represent the cut points for the quartiles listed in Table 2.

Figure 3 shows the dose–response relation between BPA and T2D. We observed a linear association among premenopausal cases only (*p*_trend_ = 0.02). Butyl phthalate metabolites were monotonically associated with an increased T2D risk among all participants, especially for those at high-exposure levels (*p*_trend_ = 0.02; see Supplemental Material, Figure S2B). DEHP and total phthalates were not significantly associated with incident T2D in this analysis.

## Discussion

In this prospective investigation among U.S. women, we found positive associations between urinary BPA and butyl phthalate concentrations and incident T2D in the NHSII participants, but not in the older NHS counterparts. The positive associations of BPA and phthalates with T2D were significant only after adjusting for BMI, although in joint analyses without further adjustment for BMI, BPA and butyl phthalates were positively associated with T2D risk among participants across BMI categories, suggesting that the positive associations may not be due entirely to the confounding by BMI. The distinct associations observed between the two cohorts could be due to differences in age distribution or menopausal status, although the role of chance cannot be excluded.

The majority of publications regarding associations of BPA/phthalates with T2D are analyses using the cross-sectional National Health and Nutrition Examination Survey (NHANES). Lang et al. (2008) reported a significant, positive association between urinary BPA concentrations and diabetes prevalence among 1,455 U.S. adults in the NHANES 2003–2004 cycle. Shankar and Teppala (2011) observed a similar association using data from NHANES 2003–2008 cycles, but an independent analysis by Silver et al. (2011) suggested this association was primarily driven by data from the 2003–2004 cycle.More recently, LaKind et al. (2012) found no associations in all NHANES cycles, probably owing to different exclusion criteria and model covariates. Data regarding phthalates were likewise exclusively from cross-sectional analyses, in which positive associations with T2D were documented for various phthalate metabolites, including DEHP metabolites (Svensson et al. 2011), MBzP (James-Todd et al. 2012), MiBP (James-Todd et al. 2012; Lind et al. 2012a), and mono-methyl phthalate (Lind et al. 2012a).

The mechanistic hypothesis underlying a link between BPA/phthalates and diabetes risk is primarily derived from animal experiments. *In vivo* and *in vitro* experiments among rodent models have shown that BPA is a ubiquitous xenoestrogen that can activate estrogen receptors (ERs), as well as some novel ER-independent signaling pathways, even at physiologically relevant low doses (Lemmen et al. 2004; Quesada et al. 2002; Wozniak et al. 2005; Zsarnovszky et al. 2005). ERs, especially ERα, when activated, regulate energy homeostasis through multiple pathways, including glucose transport, insulin secretion, and other mechanisms (Chen et al. 2009; Nadal et al. 2009). ERs are expressed in beta cells (Nadal et al. 2000), and, despite the low binding affinity of BPA to ERs *in vitro* (Kuiper et al. 1997), BPA and 17β-estradiol (E_2_) can both stimulate insulin biosynthesis and secretion by activating ERα with equal potency in mice (Alonso-Magdalena et al. 2006, 2008; Ropero et al. 2008). Administration of low-dose BPA or E_2_ (10 μg/kg) to adult mice led to chronic hyperinsulinemia, followed by insulin resistance (Alonso-Magdalena et al. 2006). Furthermore, BPA and E_2_ can also enhance insulin secretion by activating ERβ or ER-independent pathways in mouse models (Nadal et al. 2009; Soriano et al. 2012). Although these lines of evidence from animal experiments suggest interactive effects between BPA and estrogens, the extrapolation of the evidence to humans is unclear. Therefore, the stronger association of BPA with T2D risk among younger women before menopause, although biologically plausible, may also be a chance finding and should be replicated in future studies. Effects of BPA exposures on adiponectin release, oxidative stress, dyslipidemia, and other diabetes risk factors are among other potential mechanisms (Ben-Jonathan et al. 2009; Bindhumol et al. 2003; Hugo et al. 2008; Nakagawa and Tayama 2000).

Phthalates may result in increased diabetes risk through the activation of PPARs (Casals-Casas and Desvergne 2011), which are master regulators of lipid and glucose homeostasis (Evans et al. 2004). For example, *in vitro* experiments have demonstrated that MEHP induces adipogenesis by activating PPAR-γ (Feige et al. 2007), although evidence from *in vivo* studies is not entirely consistent (Feige et al. 2010). Other possible pathways include adverse effects of DEHP exposures on thyroid hormones (Gayathri et al. 2004; Sugiyama et al. 2005) and glucose metabolism (Gayathri et al. 2004; Rengarajan et al. 2007). *In vitro* studies have also showed that butylbenzyl phthalate (BBP) and its monoester metabolites, such as MBP and MBzP, were able to activate PPAR subtypes, although their effects were weaker than those for MEHP (Corton and Lapinskas 2005; Lapinskas et al. 2005). Of note, because of the between-species differences in terms of metabolism and PPAR functionality, the relevance of animal study evidence regarding phthalates and humans is unclear (Hauser and Calafat 2005). Although animal evidence may help explain the positive associations of phthalates observed in the NHSII, the lack of association in older women is not readily explained. Further, whether individual phthalate metabolites have divergent or similar metabolic effects is largely unknown, and thus caution is needed when interpreting the positive associations for total phthalates.

The present study has several caveats that deserve discussion. Our study participants were exclusively registered female nurses who are not representative of the general population, limiting the generalizability of the results to men and other ethnic groups. However, the concentrations of BPA and phthalate metabolites in these nurses substantially overlapped with those observed in the NHANES (Calafat et al. 2008; Silva et al. 2004). The homogeneity of our cohort participants in terms of socioeconomic status and universal access to health care services actually reduces confounding and improves internal validity. An important limitation of the exposure measurements is that the within-person stability of BPA concentrations over 1–3 years was quite low (ICC = 0.14), suggesting that first morning urine samples may not represent long-term exposure levels. Ideally, multiple urine samples collected over an extended period should be used to assess long-term exposures (Snijder et al. 2013) or to evaluate changes of exposures over time, but this is often not practical in large-scale epidemiologic studies. Typically, large random within-person variations and the random error of BPA measurements (as reflected by the relatively high CV) tend to diminish the ability to detect a modest association (Rothman and Greenland 1998). Likewise, misclassification of self-reported diabetes status among controls, if any, is likely to be random and thus may further attenuate the true association toward the null. In addition, the findings are also possibly due to chance, especially when divergent associations were observed in the two cohorts. In NHSII controls, we found an inverse cross-sectional relation between BPA and BMI, one the strongest risk factors for T2D. This correlation is inconsistent with the findings from the NHANES (Carwile and Michels 2011). This inverse correlation may explain the association between BPA and T2D after adjustment for BMI in the NHSII. However, adjustment for BMI minimally changed the associations between BPA and diabetes prevalence in the NHANES (Lang et al. 2008), and null associations of BPA with BMI were observed in another study (Melzer et al. 2012). Likewise, associations between phthalate metabolites and BMI were also inconsistent with the literature (Hatch et al. 2008; Lind et al. 2012a; Teitelbaum et al. 2012; Trasande et al. 2012). It is possible that dietary and lifestyle predictors of BPA and phthalate exposures may vary across populations (Braun et al. 2011; Buckley et al. 2012; Geens et al. 2012), and the cross-sectional correlations with BMI may vary accordingly. An alternate explanation for the differential associations observed between the two cohorts is that the measured pollutant concentrations may not capture the etiologically relevant exposure levels in critical time windows in the NHS because urine samples were collected at much older ages in that cohort. Last, the role of residual or unmeasured confounding cannot be excluded. Although we controlled for an array of covariates, including diet quality, some known routes of BPA and phthalate exposures, such as canned food or beverage consumption, contact with certain medical devices, and use of certain medications or consumer products (Buckley et al. 2012; Geens et al. 2012; Kelley et al. 2012), were not captured in our cohorts. Furthermore, confounding by other correlated chemicals from the same sources cannot be excluded.

## Conclusions

In this prospective study we found positive associations of urinary concentrations of BPA and certain phthalate metabolites with incident T2D among middle-aged U.S. female nurses, but not among their older counterparts. Although different distribution of age and menopausal status may underlie these divergent results, these observations may also be due to chance, residual confounding, or other biases. Therefore, our results need to be interpreted with caution, especially considering that a single urinary measurement of phthalate metabolites or BPA may not represent longer-term exposure. Clearly, our findings should be replicated in future prospective studies, and the significance of these chemicals in T2D prevention and risk prediction remains uncertain.

## Supplemental Material

(1.1 MB) PDF
