# Supplementary material for: Association of Urinary Concentrations of Bisphenol A and Phthalate Metabolites with Risk of Type 2 Diabetes: A Prospective Investigation in the Nurses’ Health Study (NHS) and NHSII Cohorts
Source: Environ Health Perspect. 2014 Mar 14;122(6):616–23. doi: 10.1289/ehp.1307201 (PMC4050512; doi:10.1289/ehp.1307201)
Supplement: (1.1 MB) PDF [file ehp.1307201.s001.pdf]

Environmental Health Perspectives doi:10.1289/ehp.1307201

Supplemental Material

Association of Urinary Concentrations of Bisphenol A and Phthalate Metabolites with Risk of Type 2 Diabetes: A Prospective Investigation in the Nurses' Health Study (NHS) and NHSII Cohorts

Qi Sun, Marilyn C. Cornelis, Mary K. Townsend, Deirdre K. Tobias, A. Heather Eliassen, Adrian A. Franke, Russ Hauser, and Frank B. Hu

Table of Contents **Page**

Methods 2

Table S1. Partial Spearman correlation coefficients among urinary concentrations of phthalate metabolites and bisphenol A (BPA) among controls, Nurses' Health Study (NHS) and NHSII 4

Table S2. Odds ratio (95% CI) of incident type 2 diabetes by quartiles of urinary concentrations of individual phthalate metabolites (µg/L), the Nurses' Health Study (NHS) and NHSII 5

Figure S1. Joint associations between urinary concentrations of bisphenol A (BPA) and butyl phthalates, Nurses' Health Study II 8

Figure S2. Pooled odds ratio (95% CI) of incident type 2 diabetes comparing extreme quartiles of individual urinary concentrations of phthalate metabolites and bisphenol A (BPA) in the Nurses' Health Study (NHS) and NHSII 9

References 12

Methods

Laboratory measurements

Two hundred μL urine was spiked with 20 μL of a mixture of isotopically labeled phthalate metabolites and BPA-$^{13}C_{12}$ to be used as internal standards followed by treatment with β-glucuronidase and sulfatase at 37°C for 90 minutes. After acidification with 50 μL glacial acetic acid extraction with 2.0 mL methyl tertiary butyl ether was performed. Half of the ether phase was dried with nitrogen for phthalate analysis while the second half of the ether phase was dried for BPA analysis. The first half of the extract was reconstituted in 125 μL 0.1% formic acid in methanol/water (1:1) for phthalates and analyzed by orbitrap-LCMS (model Exactive, Thermo Electron, Waltham, MA) (Kato et al. 2005). Mass detection was carried out in negative electrospray ionization (-ESI) mode using exact masses. Data acquisition and analysis was performed using Thermo's Xcalibur software. Detection of the analytes was set within 10 ppm of the calculated mass. Limits of detection were 0.1-1.0 ng/mL.

In a pilot study, we compared concentrations of phthalate metabolites in urine samples treated with/without using the β-glucuronidase and sulfatase to test for potential environmental contamination of samples (Blount et al. 2000). Among 44 NHS and NHSII participants, urinary concentrations of phthalates metabolites were highly correlated between the two measurements. The intraclass coefficients (ICC) were > 0.99 for MEP, MBP, MEOHP, and MBzP, > 0.96 for MEHHP and MECPP, > 0.94 for MiBP and MEHP, suggesting that the use of β-glucuronidase and sulfatase had little impact on the measurements of these chemicals. The ICC (0.82) was slightly weaker for PA measurements, although any misclassification of the true PA concentrations is likely non-differential because contamination by environmental phthalates was

unrelated with true exposures or diabetes ascertainment. Non-differential measurement errors generally attenuate true associations toward the null.

Bisphenol-A (unconjugated and conjugated metabolites) was analyzed by tandem-LCMS (model TSQ Ultra, Thermo Electron, Waltham, MA) after dansylation (Fox et al. 2011). Mass detection was carried out in positive electrospray ionization mode with spray voltage at 3.5 kV, capillary temperature 300 ^{o}C, and sheath gas (pressure 35 units) and auxiliary gas (pressure 10 units). The divert valve was set to detector from 4-15 min. Signal acquisition was performed in selected reaction monitoring (SRM) mode detecting the transition of m/z 695 > 170 for BPA and 707 > 170 for 13C12-BPA. The limit of detection was 0.05 ng/mL.

Table S1. Partial Spearman correlation coefficients[a] among urinary concentrations of phthalate metabolites and bisphenol A (BPA) among controls, Nurses' Health Study (NHS) and NHSII.

**Exposure**	**MEP**	**MEHP**	**MEHHP**	**MECPP**	**MEOHP**	**MBzP**	**MBP**	**MiBP**	**PA**	**BPA**
MEP	1.0	0.17**	0.18**	0.16**	0.18**	0.07	-	-	0.36**	0.10
MEHP	0.16**	1.0	0.69**	0.67**	0.59**	0.18**	-	-	0.26**	0.16**
MEHHP	0.19**	0.70**	1.0	0.90**	0.83**	0.17**	-	-	0.35**	0.11*
MECPP	0.18**	0.61**	0.89**	1.0	0.79**	0.15**	-	-	0.36**	0.12*
MEOHP	0.17**	0.73**	0.91**	0.84**	1.0	0.20**	-	-	0.30**	0.07
MBzP	0.09*	0.16**	0.15**	0.15**	0.18	1.0	-	-	0.07	0.11*
MBP	0.16**	0.40**	0.34**	0.34**	0.34**	0.36**	1.0	-	-	-
MiBP	0.17**	0.40**	0.33**	0.32**	0.34**	0.35**	0.98**	1.0	-	-
PA	0.47**	0.27**	0.32**	0.32**	0.37**	0.23**	0.31**	0.31**	1.0	0.21**
BPA	0.03	0.22**	0.21**	0.21**	0.26**	0.13**	0.12**	0.12**	0.18**	1.0

Abbreviations: MEP, monoethyl phthalate; MEHP, mono-(2-ethylhexyl) phthalate; MEHHP, mono-(2-ethyl-5-hydroxyhexyl) phthalate; MECPP, mono(2-ethyl-5-carboxypentyl) phthalate; MEOHP, mono-(2-ethyl-5-oxohexyl) phthalate; MBP, monobutyl phthalate; MiBP, mono-isobutyl phthalate; MBzP, monobenzyl phthalate; PA, phthalic acid; and BPA, bisphenol A.

[a]Adjusted for age at urine sample collection (yr), time of blood draw, race (white or not), fasting status (yes, no), and urinary creatinine levels (µg/dL). Coefficients in the lower left section were for the NHSII (n = 577), and those in the upper section were for the NHS (n = 393 except for PA n = 249).

*P < 0.05. **P < 0.01.

Table S2. Odds ratio (95% CI) of incident type 2 diabetes by quartiles of urinary concentrations of individual phthalate metabolites (μg/L), the Nurses' Health Study (NHS) and NHSII.

**Model**	**Quartile 1 (lowest)**	**Quartile 2**	**Quartile 3**	**Quartile 4 (highest)**	**P for trend**
**Nurses' Health Study**					
MEP					
Median (Range)	16.7 (1.5, 33.3)	54.1 (33.4, 81.8)	123.8 (81.9, 186.4)	421.2 (187.3, 9596.5)	
Case/control	93/98	111/98	105/99	85/98	
Model 1[a]	1	1.17 (0.79, 1.74)	1.07 (0.71, 1.61)	0.80 (0.52, 1.23)	0.11
Model 2[b]	1	1.12 (0.72, 1.75)	0.98 (0.62, 1.56)	0.70 (0.43, 1.14)	0.06
Model 3[c]	1	1.13 (0.69, 1.84)	0.98 (0.60, 1.61)	0.72 (0.43, 1.20)	0.09
MEHP					
Median (Range)	1.4 (0.2, 2.4)	3.7 (2.4, 4.7)	6.3 (4.7, 9.3)	16.7 (9.4, 502.0)	
Case/control	95/98	104/97	89/99	106/99	
Model 1[a]	1	1.04 (0.68, 1.59)	0.83 (0.53, 1.29)	0.94 (0.59, 1.51)	0.80
Model 2[b]	1	1.10 (0.69, 1.77)	0.80 (0.48, 1.33)	1.16 (0.68, 1.99)	0.53
Model 3[c]	1	1.23 (0.73, 2.06)	0.85 (0.50, 1.47)	1.29 (0.72, 2.30)	0.39
MEHHP					
Median (Range)	8.6 (0.1, 12.1)	16.2 (12.1, 22.3)	30.3 (22.3, 43.6)	88.6 (44.0, 2577.5)	
Case/control	97/98	84/99	91/98	122/98	
Model 1[a]	1	0.82 (0.54, 1.26)	0.87 (0.58, 1.32)	1.13 (0.72, 1.75)	0.22
Model 2[b]	1	0.93 (0.57, 1.51)	0.85 (0.53, 1.35)	1.27 (0.76, 2.11)	
Model 3[c]	1	0.87 (0.51, 1.47)	0.80 (0.48, 1.31)	1.22 (0.71, 2.09)	0.14
MECPP					
Median (Range)	11.2 (0.1, 14.9)	20.7 (15.1, 26.5)	37.1 (27.1, 56.2)	102.0 (56.4, 2041.0)	
Case/control	64/99	98/99	124/98	108/97	
Model 1[a]	1	1.63 (1.02, 2.59)	1.94 (1.23, 3.05)	1.75 (1.06, 2.91)	0.33
Model 2[b]	1	2.05 (1.21, 3.48)	2.07 (1.23, 3.49)	2.12 (1.20, 3.77)	0.24
Model 3[c]	1	2.26 (1.28, 3.97)	1.94 (1.11, 3.38)	2.30 (1.24, 4.29)	0.18
MEOHP					
Median (Range)	5.7 (0.1, 8.5)	10.9 (8.5, 14.6)	18.9 (14.6, 28.1)	55.7 (28.2, 3104.0)	
Case/control	98/98	85/99	93/98	118/98	
Model 1[a]	1	0.84 (0.56, 1.24)	0.90 (0.59, 1.36)	1.06 (0.68, 1.65)	0.45
Model 2[b]	1	0.81 (0.52, 1.27)	0.83 (0.51, 1.33)	1.13 (0.68, 1.86)	0.28
Model 3[c]	1	0.75 (0.49, 1.37)	0.82 (0.49, 1.37)	1.21 (0.70, 2.09)	0.15
MBzP					
Median (Range)	3.5 (0.04, 5.2)	7.2 (5.3, 9.4)	13.4 (9.6, 18.3)	31.8 (18.4, 1415.5)	
Case/control	97/98	93/99	95/99	109/98	
Model 1[a]	1	0.89 (0.59, 1.35)	0.84 (0.56, 1.28)	0.91 (0.57, 1.43)	0.83
Model 2[b]	1	1.02 (0.64, 1.63)	0.86 (0.54, 1.37)	0.85 (0.51, 1.41)	0.45
Model 3[c]	1	0.91 (0.55, 1.51)	0.85 (0.51, 1.40)	0.82 (0.48, 1.43)	0.54

**Model**	**Quartile 1 (lowest)**	**Quartile 2**	**Quartile 3**	**Quartile 4 (highest)**	**P for trend**
Phthalic acid					
Median (Range)	31.9 (0.9, 41.0)	55.5 (41.3, 69.7)	86.7 (70.1, 112.9)	197.8 (114.9, 1846.7)	
Case/control	48/62	60/62	62/62	80/63	
Model 1[a]	1	1.22 (0.72, 2.06)	1.15 (0.67, 1.99)	1.45 (0.81, 2.60)	0.24
Model 2[b]	1	1.34 (0.74, 2.42)	1.26 (0.68, 2.33)	1.57 (0.82, 3.02)	0.25
Model 3[c]	1	1.37 (0.71, 2.64)	1.23 (0.63, 2.42)	1.61 (0.79, 3.29)	0.26
**Nurses' Health Study II**					
MEP					
Median (Range)	18.7 (1.5, 43.1)	70.2 (43.2, 107.2)	153 (108.6, 234.2)	430.6 (234.3, 10374.2)	
Case/control	173/144	161/144	127/145	116/144	
Model 1[a]	1	0.93 (0.67, 1.29)	0.56 (0.39, 0.80)	0.87 (0.61, 1.24)	0.74
Model 2[b]	1	0.97 (0.66, 1.41)	0.60 (0.39, 0.91)	0.90 (0.60, 1.35)	0.85
Model 3[c]	1	1.00 (0.60, 1.67)	0.46 (0.26, 0.82)	0.91 (0.54, 1.54)	0.97
MEHP					
Median (Range)	2.1 (0.2, 3.6)	4.9 (3.6, 6.6)	8.6 (6.6, 11.6)	18.3 (11.6, 433.8)	
Case/control	163/134	147/133	120/137	109/135	
Model 1[a]	1	0.87 (0.63, 1.20)	0.63 (0.44, 0.89)	0.56 (0.39, 0.80)	0.001
Model 2[b]	1	0.83 (0.57, 1.21)	0.86 (0.57, 1.29)	0.62 (0.41, 0.95)	0.03
Model 3[c]	1	1.15 (0.71, 1.86)	0.95 (0.56, 1.62)	0.73 (0.42, 1.28)	0.15
MEHHP					
Median (Range)	10.6 (0.1, 15.0)	19.9 (15.1, 27.1)	35.4 (27.3, 49.3)	86.2 (49.3, 1071.0)	
Case/control	109/144	148/144	144/145	176/144	
Model 1[a]	1	1.34 (0.96, 1.88)	1.29 (0.90, 1.84)	1.59 (1.09, 2.31)	0.05
Model 2[b]	1	1.34 (0.91, 1.97)	1.37 (0.91, 2.06)	1.71 (1.11, 2.64)	0.04
Model 3[c]	1	1.80 (1.08, 3.00)	1.65 (0.97, 2.80)	1.97 (1.12, 3.48)	0.11
MECPP					
Median (Range)	14.2 (0.1, 19.2)	26.2 (19.3, 34.6)	44.3 (34.7, 62.6)	101.3 (62.8, 1169.6)	
Case/control	96/144	154/144	151/145	176/144	
Model 1[a]	1	1.63 (1.14, 2.33)	1.65 (1.13, 2.41)	1.93 (1.28, 2.90)	0.02
Model 2[b]	1	1.48 (0.98, 2.23)	1.63 (1.04, 2.55)	2.02 (1.26, 3.24)	0.01
Model 3[c]	1	1.70 (0.99, 2.93)	1.61 (0.91, 2.85)	2.05 (1.09, 3.84)	0.10
MEOHP					
Median (Range)	7.0 (0.1, 9.9)	14.0 (10, 18.4)	23.9 (18.4, 34.1)	51.4 (34.6, 854.0)	
Case/control	107/144	162/144	156/145	152/144	
Model 1[a]	1	1.50 (1.06, 2.13)	1.39 (0.97, 2.00)	1.33 (0.90, 1.95)	0.63
Model 2[b]	1	1.48 (0.99, 2.21)	1.56 (1.02, 2.37)	1.50 (0.96, 2.34)	0.29
Model 3[c]	1	1.93 (1.13, 3.29)	2.12 (1.21, 3.71)	1.71 (0.95, 3.06)	0.41

Model	Quartile 1 (lowest)	Quartile 2	Quartile 3	Quartile 4 (highest)	P for trend
MBzP					
Median (Range)	8.8 (0.04, 13)	17.2 (13.0, 23.2)	33.3 (23.2, 47.3)	87.1 (47.3, 766.6)	
Case/control	142/144	160/144	140/145	135/144	
Model 1[a]	1	1.08 (0.77, 1.50)	0.87 (0.61, 1.23)	0.80 (0.55, 1.16)	0.12
Model 2[b]	1	0.93 (0.63, 1.37)	0.88 (0.59, 1.32)	0.83 (0.54, 1.27)	0.42
Model 3[c]	1	0.85 (0.50, 1.44)	1.08 (0.62, 1.86)	1.14 (0.65, 2.01)	0.44
MBP					
Median (Range)	13.9 (0.2, 19.6)	26.3 (19.6, 32.2)	39.4 (32.2, 49.2)	78.1 (49.6, 3959)	
Case/control	143/144	144/144	110/145	180/144	
Model 1[a]	1	0.97 (0.69, 1.37)	0.70 (0.48, 1.02)	1.14 (0.78, 1.67)	0.25
Model 2[b]	1	1.06 (0.71, 1.57)	0.81 (0.53, 1.26)	1.53 (0.98, 2.40)	0.02
Model 3[c]	1	1.53 (0.90, 2.61)	1.18 (0.67, 2.09)	3.16 (1.69, 5.92)	0.0003
MiBP					
Median (Range)	9.5 (0.2, 13.9)	18.3 (14.0, 22.7)	27.5 (22.7, 33.9)	52.1 (34.2, 2599.6)	
Case/control	147/144	142/143	109/146	179/144	
Model 1[a]	1	0.92 (0.65, 1.29)	0.66 (0.45, 0.97)	1.09 (0.74, 1.61)	0.34
Model 2[b]	1	0.94 (0.63, 1.40)	0.76 (0.49, 1.18)	1.36 (0.86, 2.14)	0.07
Model 3[c]	1	1.28 (0.76, 2.13)	1.12 (0.62, 2.02)	2.67 (1.44, 4.95)	0.001
Phthalic acid					
Median (Range)	35.9 (0.9, 51.8)	64.5 (51.9, 85.3)	113.6 (85.4, 156.8)	225.5 (158.6, 2295.3)	
Case/control	104/144	131/144	167/145	175/144	
Model 1[a]	1	1.31 (0.91, 1.88)	1.63 (1.12, 2.36)	1.69 (1.15, 2.50)	0.02
Model 2[b]	1	1.47 (0.96, 2.23)	1.97 (1.27, 3.07)	1.79 (1.14, 2.82)	0.05
Model 3[c]	1	1.56 (0.90, 2.71)	2.13 (1.19, 3.79)	1.77 (0.97, 3.23)	0.19

Abbreviations: MEP, monoethyl phthalate; MEHP, mono-(2-ethylhexyl) phthalate; MEHHP, mono-(2-ethyl-5-hydroxyhexyl) phthalate; MECPP, mono(2-ethyl-5-carboxypentyl) phthalate; MEOHP, mono-(2-ethyl-5-oxohexyl) phthalate; MBP, monobutyl phthalate; MiBP, mono-isobutyl phthalate; MBzP, monobenzyl phthalate; PA, phthalic acid; and BPA, bisphenol A.

[a]Model 1 was adjusted for the matching factors, including age at urine sample collection (yrs), race (white or not), fasting status (yes, no), time of blood drawing, and menopausal status and use of hormone replacement therapy (NHSII only), and urinary creatinine levels (mg/dL). [b]Based on model 1, model 2 was further adjusted for smoking status (current smoker, past smoker, non-smoker), postmenopausal hormone use (yes, no; NHS only), oral contraceptive use (never used, past user, current user; NHSII only), physical activity (METs-hr/week), alcohol use (abstainer, < 5.0 g/day, 5.0-14.9 g/day, ≥ 15.0 g/day), family history of diabetes (yes, no), history of hypercholesterolemia or hypertension (yes, no), and alternative Health Eating Index score. [c]Based on model 2, model 3 was further adjusted for body mass index (< 25.0 kg/m^2, 25.0-27.4 kg/m^2, 27.5-29.9 kg/m^2, 30.0-32.4 kg/m^2, ≥ 32.5 kg/m^2, and missing category).

Figure S1. Joint associations between urinary concentrations of bisphenol A (BPA) and butyl phthalates, Nurses' Health Study II.

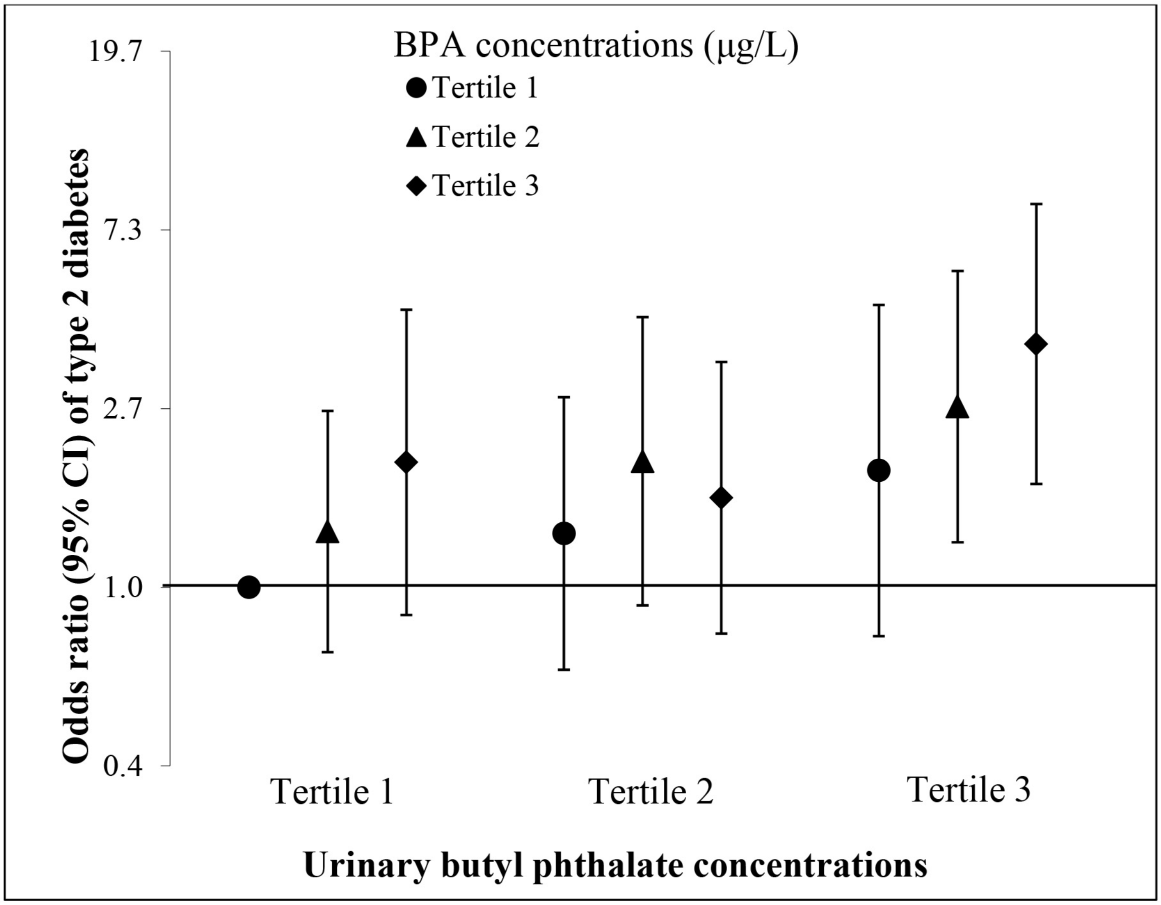

Conditional logistic regression model was adjusted for the same set of covariates as in the Model 3, Table 2.

Figure S2. Odds ratio (95% CI) of incident type 2 diabetes by urinary concentrations of phthalate metabolites.

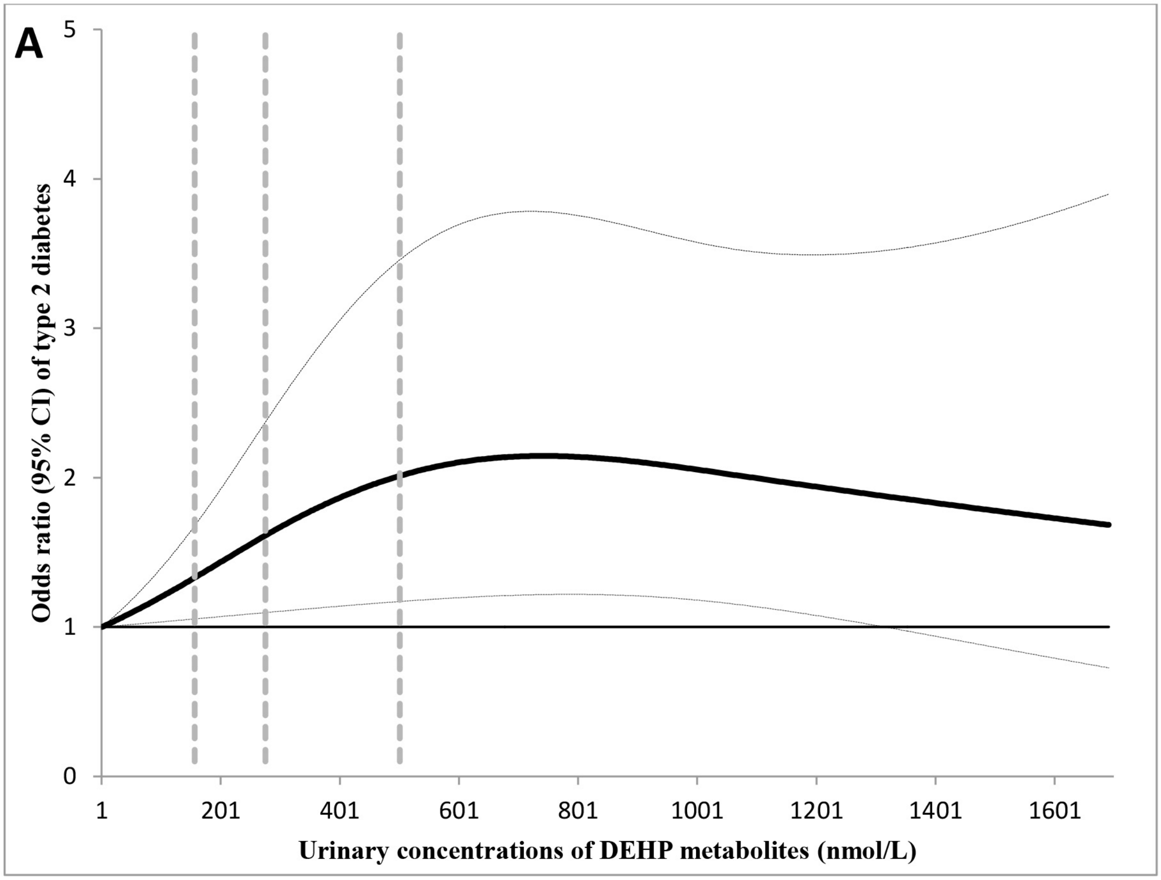

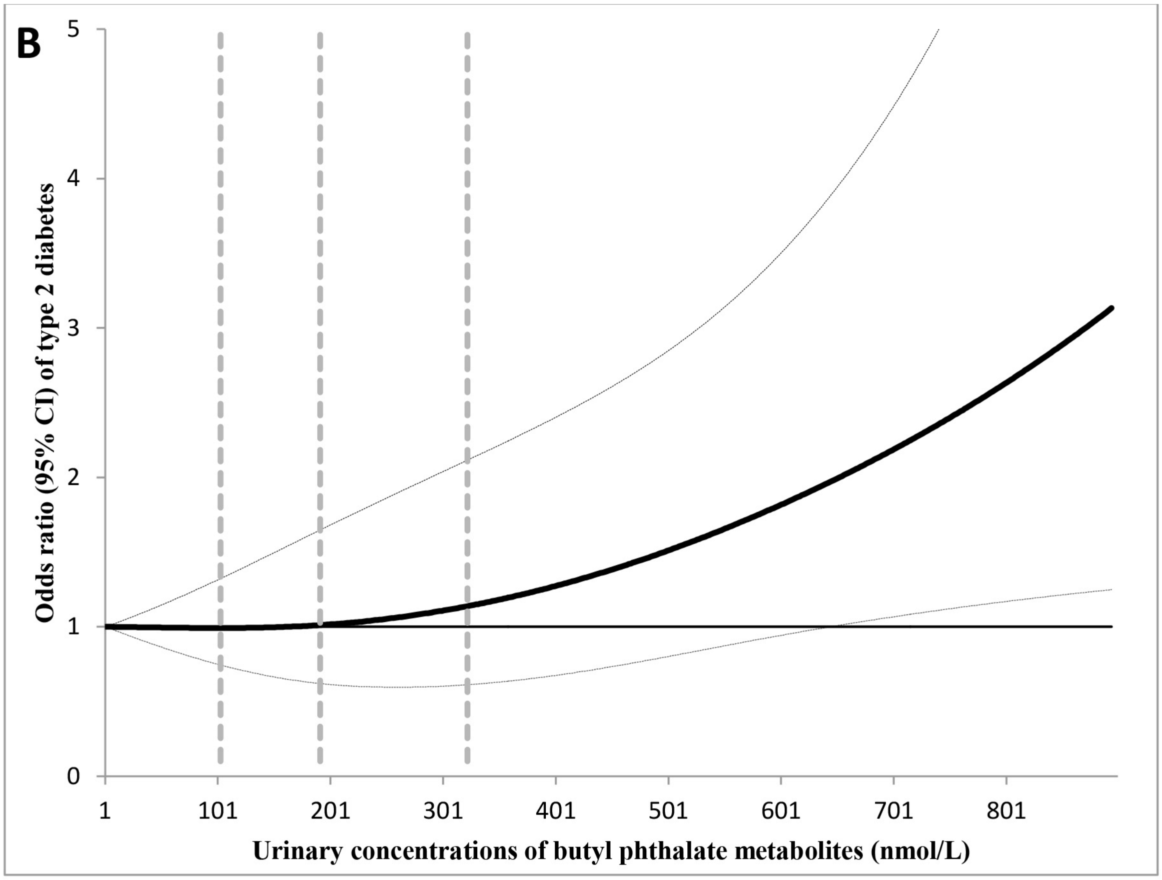
B
5
4
3
2
1
0
Odds ratio (95% CI) of type 2 diabetes
1
101
201
301
401
501
601
701
801
Urinary concentrations of butyl phthalate metabolites (nmol/L)

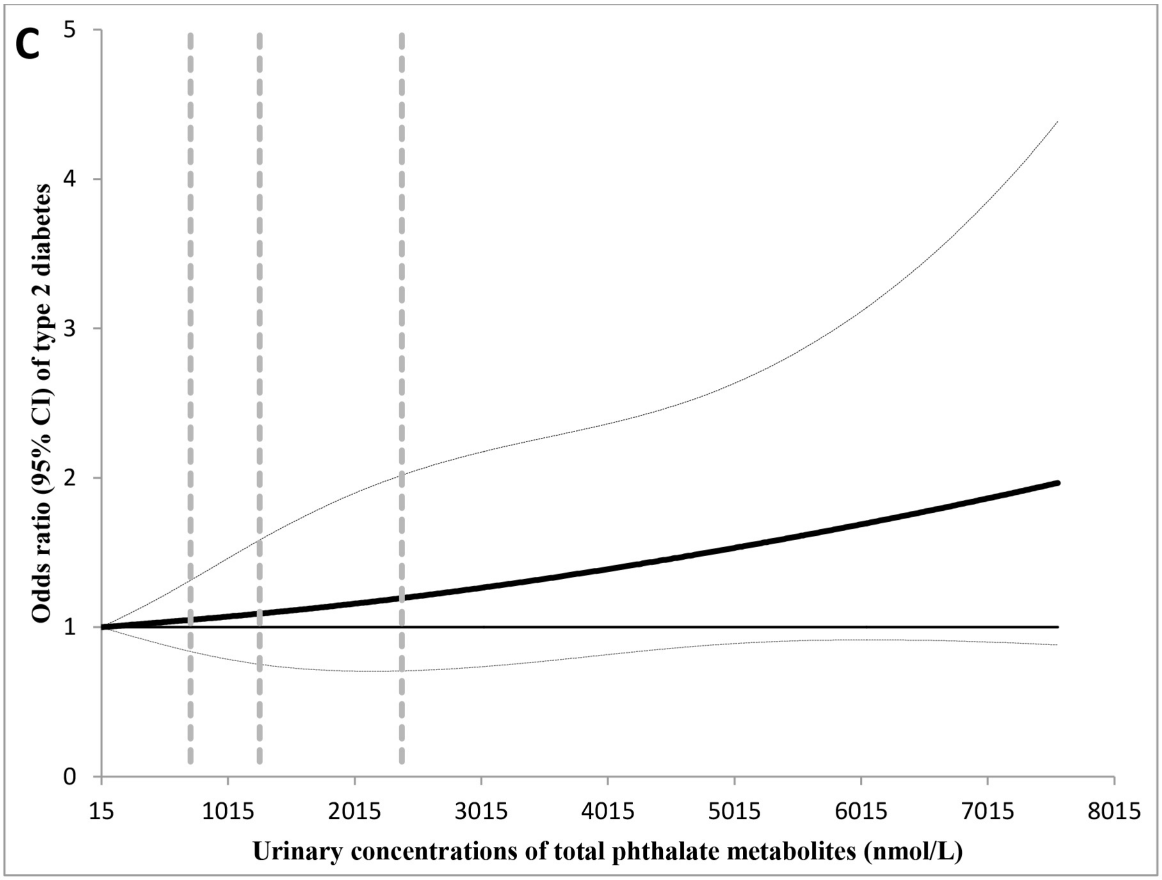

Study participants with the highest 5% of phthalate concentrations were excluded to minimize potential impact of outliers. Multivariate conditional logistic regression models were adjusted for the same set of covariates for model 3 in Table 2. Solid lines are ORs and dashed lines are 95% CIs. The dotted vertical lines represent the cut-off points for making quartiles listed in Table 2. A, DEHP metabolites; B, Butyl phthalate metabolites; C, total phthalate metabolites.

References

Blount BC, Silva MJ, Caudill SP, Needham LL, Pirkle JL, Sampson EJ, et al. 2000. Levels of seven urinary phthalate metabolites in a human reference population. Environ Health Perspect 108(10):979-982.

Fox SD, Falk RT, Veenstra TD, Issaq HJ. 2011. Quantitation of free and total bisphenol A in human urine using liquid chromatography-tandem mass spectrometry. J Sep Sci 34(11):1268-1274.

Kato K, Silva MJ, Needham LL, Calafat AM. 2005. Determination of 16 phthalate metabolites in urine using automated sample preparation and on-line preconcentration/high-performance liquid chromatography/tandem mass spectrometry. Anal Chem 77(9):2985-2991.
